# Protein Intake during the First Two Years of Life and Its Association with Growth and Risk of Overweight

**DOI:** 10.3390/ijerph15081742

**Published:** 2018-08-14

**Authors:** Minghua Tang

**Affiliations:** Section of Nutrition, Department of Pediatrics, University of Colorado School of Medicine, Aurora, CO 80045, USA; minghua.tang@ucdenver.edu; Tel.: +1-303-724-3248

**Keywords:** protein, complementary feeding, growth, the gut microbiome

## Abstract

Growth patterns early in life could exert a long-term impact on overweight and obesity development. Among all potential manipulative factors, infant diet is one of the most influential and could affect growth and subsequent health status during adolescence and adulthood. Dietary protein, as an important macronutrient in infants’ diet, has been of special interest to researchers. Compared with human milk, infant formula tends to have a higher protein content and is associated with greater weight gain and later-in-life obesity risk. However, the effect of protein from other sources on infant growth trajectories during complementary feeding is not clear. Emerging research suggests that meat protein during early complementary feeding promotes linear growth while not increasing risk of overweight compared with dairy protein; and the gut microbiota might be a mediator between protein quality and growth trajectories. This review addresses the current knowledge of protein intake from birth to 24 months and its relationship with growth and risk of overweight.

## 1. Introduction

The period from birth to 24 months covers two-thirds of the “First 1000 days” critical window and is linked to the development of overweight and obesity in the long term [1]. Emerging epidemiologic evidence suggests that rapid weight gain early in life predisposes to later obesity development. Preventative post-natal interventions from birth to 24 months need to focus on dietary intake, not only because weight gain is highly dependent upon infants’ diet, which consists of breastmilk/formula and complementary foods, but also because it can be modified. Infants are exposed to various food and protein sources beyond breastmilk/formula (e.g., dairy protein) during complementary feeding (~6 to 24 months) [2], which is a critical window for long-term food preference and habits [3]. Thus, evidence-based complementary feeding guidance could not only help prevent later overweight and obesity, but also establish healthy life-long dietary patterns [4]. Among all dietary variables, protein is considered a key player in infant weight regulation based on both interventional and observational studies [5,6]. However, the effects of protein intake on growth trajectories from birth to 24 months and the underlying mechanisms are not well-understood.

Given the current obesity rates in westernized settings [7], a better understanding of potentially modifiable risk factors underpinning excessive weight and adiposity gain early in life is urgently needed. The Dietary Guidelines for Americans (DGA) generated by the United States Department of Agriculture and the US Department of Health and Human Services are published every 5 years and provide essential guidance for consumers, researchers, and health professionals. However, the current DGA has very limited guidance for infants and young children from birth to 24 months because of the limited amount of high-quality research available. The DGA B-24 (birth to 24 months) project has been initiated by the DGA committee to evaluate evidence and encourage publications in high-priority topics in order to include this age group in future DGAs [8]. In particular, differences in protein intake (e.g., total amount and source) on growth is a top-priority topic for infants from 6 to 12 months [8]. A similar emphasis has also been proposed by scientists in Europe [9]. This narrative review will discuss up-to-date research (April, 2018) on the relation between protein intake and growth and risk of overweight in infants and young children in developed settings as well as factors that could potentially influence this relationship, such as certain essential amino acids and the gut microbiome.

## 2. Effect of Protein from Breastmilk and Infant Formula

Breastmilk is the ideal sole source of nutrients for young infants, and the World Health Organization (WHO) recommends infants to be exclusively breastfed for the first 6 months of life [10]. In terms of protein content, breastmilk is whey-protein dominant and α-lactalbumin accounts for over 40% of the whey protein in breastmilk [11]. It is well-documented that α-lactalbumin is protective against common pathogens, such as *Escherichia coli* [12]. In the situation where breastfeeding is not possible, feeding with a suitable breastmilk substitute, such as cow and goat-milk-based formulas, is recommended [13]. Cow-milk-based infant formula is widely used in developed settings as a substitute for breastmilk. However, formula-fed infants tend to have greater weight gain than breastfed infants, which has been demonstrated in multiple observational studies [14,15,16]. Most standard cow-milk-based infant formula tend to have a higher protein content than breastmilk (~2.2 g/100 kcal versus ~1.5 g/100 kcal). This discrepancy has been considered the key contributor to the greater weight gain observed in formula-fed infants based on the “early protein hypothesis” [17]. This hypothesis suggests that a higher protein intake via infant formula increases certain circulating essential amino acids, which stimulate the secretion of insulin and insulin-like-growth factor 1 (IGF-1). These anabolic hormones then promote weight and fat gain in formula-fed infants. This hypothesis was tested in a large-scale, randomized controlled trial [6] that compared iso-caloric infant formula with high- (2.9 g/100 kcal) and low-protein (1.7 g/100 kcal) contents from birth to 12 months. Results showed that infants who consumed the low-protein formula had a growth trajectory similar to breastfed infants. However, infants who consumed the high-protein formula had similar length gain but a more rapid weight gain, which led to a 0.20 SD higher weight-for-length Z score (WLZ), a parameter of overweight risk, at 12 months [6]. These high-protein intake-induced growth patterns also persisted at school age [18]. Similar findings were demonstrated in another study [19]. Another potential possibility of the protective effect of breastmilk on risk of overweight is the different amino acid composition between breastmilk and infant formula, in particular, glutamate content [20]. Some research showed that the consumption of free glutamate, which is higher in breastmilk than in infant formula, downregulates an infant’s appetite and energy intake [20]. However, this may not be associated with infant growth [21].

These findings suggest that protein quantity in infant formula is positively associated with increased risks of overweight and obesity, and these effects may have a long-term impact beyond the first two years of life. Indeed, the current recommendation by the European Society for Paediatric Gastroenterology, Hepatology, and Nutrition (ESPGHAN) Committee on Nutrition is to limit protein intake to 15% of total energy for infants and toddlers [22]. However, by nature of design, most of these studies focused on dairy protein, because it is the primary protein source in infant formula. The effects of protein quality (e.g., protein from other sources) on growth trajectories and risk of overweight, which is critical for complementary feeding guidance, remain unclear.

## 3. Effect of Protein from Complementary Foods beyond Liquid Substitutes

Complementary foods in the present discussion include solid foods consumed from ~6 to 24 months, besides breastmilk, formula, cow’s milk, or other liquid substitutes [2]. High-quality research, especially randomized controlled trials, focusing on identifying and characterizing modifiable risk factors to prevent overweight and obesity during complementary feeding is quite limited. Current literature on protein quantity and growth during complementary feeding is primarily observational [23]. One epidemiologic study in the U.K. showed that a higher proportion of energy intake from protein at 21 months was associated with higher body mass index (BMI) and weight, but not height [23]. Similar results were found in other studies [5,24], suggesting the overall weight-accelerating effect of protein quantity during complementary feeding on growth and weight gain, which is consistent with findings of protein in breastmilk/formula [6].

Evaluating the effect of protein quality (i.e., source) on growth and risk of overweight is more complicated, and findings have been mixed. Important dietary sources of protein during complementary feeding include dairy (e.g., milk, yogurt, and cheese), meat (e.g., beef, pork, poultry), fish, and plant-based protein. As recommended by the WHO, meat is considered an excellent source of high-quality protein and micronutrients and should be consumed by infants who are consuming solid foods [25,26]. The effect of meat consumption on infant growth has been examined in various settings. One double-blinded randomized controlled trial compared a high-meat versus a low-meat diet on iron status (primary outcome) and growth from 4 to 11 months of age in Germany [27]. Protein intakes were 26% versus 18% of total energy between groups. Results showed no difference of weight or length change between groups. This might be due to: (1) the relatively small difference of protein or meat content between groups; (2) the relatively high protein intakes for both groups. Another randomized controlled trial in Denmark also found that consuming a low-protein, low-meat (10 g/d meat) versus a high-protein, high-meat (27 g/d meat) complementary diet had a comparable impact on growth from 8 to 10 months [28], although the duration of the study is relatively short and the total protein intake was not significantly different between groups. One study by Tang et al. [29] compared a high-protein, meat-based complementary diet (2.9 g/kg/d; 17% total energy) versus a low-protein, cereal-based complementary diet (1.4 g/kg/d, 9% total energy) on growth from 5 to 9 months in the U.S., and results showed that the high-protein, meat-based diet promoted weight and length gain. Specifically, the parameter of linear growth length-for-age Z score (LAZ) increased in the high-protein group and decreased in the low-protein group. Another larger trial in China [30] randomized infants to receive a daily supplement of either meat or cereal from 6 to 18 months and found that infants who consumed the meat supplement had a greater length gain and a smaller decrease of LAZ. There are also some observational trials that looked at the association between meat intake and growth during infancy (0 to 12 months) and early childhood. Several papers were published based on the Childhood Asthma Prevention Study (CAPS) in Australia. Results showed that children in the highest quintile of meat intake were significantly taller and heavier at 18 months and this association persisted at 8 [31] and 11 years of age [32]. In particular, meat intake at 18 months was positively associated with an early and persistent BMI (75th percentile at 2 years and 95th percentile at 11 years) from 0 to 11 years in boys [32]. However, there was no statistical difference of protein intake (% total energy) between boys who had a normal BMI trajectory (15.46% protein) versus those who had an early and persistent BMI (16.78% protein) or late increase BMI (15.52% protein) [32]. The comparable protein intake between BMI categories suggested that meat may have an independent influence on growth and BMI [32]. Overall, these findings suggested that meat intake in early childhood may affect growth, although the results are mixed. Most studies found no effect of meat on growth but some found that meat promotes weight and/or length gain. The majority of these studies had the primary outcome as iron status because meat is a good source of highly bioavailable iron. Future randomized controlled trials are needed to directly assess the effect of meat on infant growth, with outcomes beyond weight and length, such as body composition (e.g., visceral versus subcutaneous fat).

There are not many studies on the influence of dairy-based protein on infant and child growth and the results have been mixed. In the CAPS study, intakes of dairy foods (% total energy) at 18 months were negatively associated with adiposity at 8 years, while meat intake (% total energy) at 18 months was positively associated with adiposity at 8 years [31]. In contrast, Gunther et al. [33] observed that dairy protein intake at 12 months was associated with BMI at 7 years, while protein from meat, plants, or cereal did not have the same effect. Indeed, a recent randomized controlled trial [34] directly compared a meat- or dairy-based diet on growth from 5 to 12 months. Participants were matched between groups for sex and race; maternal height and BMI were included as covariates. Both groups consumed the same amount of protein at 3 g/kg/d or ~15% of total energy during the intervention. Results showed that infants who consumed a meat-based complementary diet increased LAZ while those who consumed a dairy-based complementary diet decreased LAZ, and both groups gained a comparable amount of weight. The difference in linear growth between groups led to a significant increase of weight-for-length Z score (WLZ), a parameter of overweight risk, in the dairy group. Overall, it appears that even within common animal sources of protein (i.e., dairy versus meat), the influence on growth could still be different.

Although it appears that a higher amount of protein intake is associated with greater weight gain and higher BMI in the long term, the effect of protein from various sources on growth is not clear. Most of the current literature focusing on protein source is observational. Limited randomized controlled trials showed that meat and dairy may have different influences on growth trajectories and weight gain during complementary feeding. Future high-quality research with mechanistic investigations is urgently needed to directly assess protein sources (i.e., dairy versus meat versus plant) on growth trajectories.

## 4. Possible Mechanisms

According to the early protein hypothesis, higher protein intake increases the concentrations of circulating amino acids, such as the branched-chain amino acids (BCAA). These amino acids could increase the secretion of insulin and IGF-1 [9], and result in rapid weight gain and body fat deposition. Indeed, the European Childhood Obesity Trial Study Group showed that infants who consumed a high-protein formula (2.9 g/100 kcal) had significantly higher serum concentrations of BCAA and other essential amino acids compared with infants who consumed a low-protein formula (1.7 g/kcal) at 6 months. Further, total IGF-1 was significantly associated with growth until 6 months [35]. IGF-1 is one of the most copious growth factors in human bone [36] and greatly influences linear growth (LAZ). Both animal [37] and human studies [38] showed that IGF-1 is associated with bone growth. The recent randomized controlled trial [34] that compared meat versus dairy on infant growth found a similar increase of serum IGF-1 in both diet groups, probably due to the comparable protein quantity between groups. However, the distinctive growth trajectories between the meat and dairy groups indicated that there might be other mediators linking protein intake and risk of overweight besides IGF-1.

The two-way obesity model, which includes the interaction between the human body and the environment, has been revised to include gut microbiota as a third player [39]. Diet strongly affects human health, partly by modulating gut microbiota [40]. Michaelson et al. in a recent review [2] called for more research on the relation between the complementary diet and the gut microbiota because current research is quite limited. One recent observational study monitored infant gut microbiota longitudinally and found that the two most major determinants of the gut microbial composition in infants are breastfeeding duration and the complementary diet [41]. Specifically, the major driver of gut microbiota changes during late infancy is the consumption of protein and fiber in complementary foods [41]. For example, the consumption of meats, cheese, and Danish rye bread increases the alpha diversity of the gut microbiota [41]. Emerging research also suggests a potential causal relation between the gut microbiota and infant growth. Two recent studies [42,43] identified bacterial species whose proportional representation defines a healthy gut microbiota during the first year of life. Specifically, deviation from the normal gut microbiota resulted in “immature” gut microbiota and growth impairment in Malawian infants [43]. Moreover, transplanting gut microbiota from stunted infants to germ-free mice also transmitted impaired growth phenotypes [43]. Some specific growth-discriminatory strains include *Ruminococcus*, *Faecalibacterium*, *Bifidobacterium*, and *Roseburia* [43]. These findings suggest that the gut microbiota may be causally related to growth regulation. However, this current research [42,43] was primarily performed in developing countries and focused on growth impairment and malnutrition. More investigations are needed in nutrient-adequate settings (e.g., developed countries) and in relation to promoting optimal growth while reducing risk of overweight.

When studying certain nutrients (e.g., protein) in the form of whole foods, other compounds in these whole foods may contribute to the outcome measures and reduce the internal validity of the findings. For example, red meat is also an excellent source of highly bioavailable iron, which is an essential micronutrient for infant growth and neurodevelopment [44]. Iron is also an indispensable nutrient for many pathogenic bacteria in the gut, including *Salmonella* spp., *Shigella* spp., and pathogenic *E. coli* [45]. Several studies have shown that iron supplementation during late infancy increases the abundance of potential pathogens and inflammation [46,47]. Thus, iron in meat may also affect growth and the gut microbiota in infants. Meat and dairy foods also differ in fat composition and calcium and vitamin D contents; and future research needs to consider these potential variants when interpreting the results.

## 5. Conclusions

The impact of protein on infant growth and overweight needs to be evaluated from both the quantity and quality perspectives. Although most studies demonstrated the positive association between protein quantity and weight gain, the differential effects of protein from various common sources, such as meat, dairy, and plants, are still unclear. Current research suggests that dairy protein has a strong effect on growth and risk of overweight, but effects from meat are still unclear, and the findings are mixed. Emerging research showed that meat may not have the same weight-accelerating effect as dairy protein does. Possible mechanisms of the association between protein and growth include the regulation of hormones, such as IGF-1, and the gut microbiome. More research is needed to directly compare various protein sources on growth trajectory and risk of overweight in infants and young children, including outcomes beyond just weight and length, such as body composition and the gut microbiome, before definitive dietary recommendations to promote optimal growth and reduce risk of overweight can be made.
